# The Combined Effect of pH and Temperature on the Survival of *Salmonella enterica* Serovar Typhimurium and Implications for the Preparation of Raw Egg Mayonnaise

**DOI:** 10.3390/pathogens8040218

**Published:** 2019-11-04

**Authors:** Thilini Piushani Keerthirathne, Kirstin Ross, Howard Fallowfield, Harriet Whiley

**Affiliations:** Environmental Health Group, College of Science and Engineering, Flinders University, GPO BOX 2100, Adelaide 5001, Australia; kirstin.ross@flinders.edu.au (K.R.); howard.fallowfield@flinders.edu.au (H.F.); harriet.whiley@flinders.edu.au (H.W.)

**Keywords:** resuscitation, mayonnaise, salmonellosis, gastroenteritis, public health, chicken eggs

## Abstract

Raw egg products are often associated with salmonellosis. The Australian guidelines recommend raw egg mayonnaise to be prepared and stored under 5 °C and adjusted to a pH less than 4.6 or 4.2. Despite these guidelines, a significant amount of salmonellosis outbreaks are recorded annually in Australia. The aim of this study was to investigate the effect of pH and temperature on the survival of *Salmonella* Typhimurium (ST) in peptone water (PW) and mayonnaise. The pH of PW and mayonnaise was adjusted to 4.2, 4.4 and 4.6 using acetic acid and vinegar, respectively. The PW and mayonnaise were inoculated with ST and incubated at 37 °C, 23 °C, and 4 °C. The survival of *Salmonella* was determined using the drop plate method. Survival was significantly (*p* < 0.05) improved at 4 °C. In both mayonnaise and PW, following 24 h, there was no ST growth at pH 4.2. Resuscitation of ST was rapidly observed at 4 °C while complete inactivation was observed at 37 °C at pH 4.2, 4.4, and 4.6 in both PW and mayonnaise. Lower temperatures protected ST from the bactericidal effect of low pH. “The preparation of mayonnaise at pH 4.2 or less and incubating it at room temperature for at least 24 h could reduce the incidence of salmonellosis”.

## 1. Introduction

Nontyphoidal salmonellosis is a gastrointestinal foodborne illness affecting public health [1]. In Australia, the incidence of salmonellosis has been increasing over the last decade [2] with the primary source of outbreaks identified as raw eggs and raw egg products [3,4]. According to Patrick et al. [5], 80% of the 371 recorded outbreaks from 1985 to 1999 were related to eggs or egg products. Similarly, in South Australia, from March 2017 to July 2018, salmonellosis outbreaks were predominantly recorded due to eggs and raw egg products [6]. *Salmonella enterica* serovar Typhimurium (ST) is one of the most commonly identified [7,8].

Mayonnaise is a methodically prepared, semi-solid sauce, which is a combination of raw eggs, vinegar, oil, and spices [9,10]. The most common control mechanisms for *Salmonella* in raw egg products involve various combinations of time, temperature, and pH [11]. According to the current guidelines in different states of Australia (New South Wales, Victoria, and South Australia), raw egg products should be stored under 5 °C and the pH should be 4.2 or less than 4.6. Smittle [12] states that as factory-made mayonnaise is produced using pasteurized eggs, it isn’t a threat to public health. On the other hand, on-site manufactured mayonnaise has been linked with several salmonellosis outbreaks [13,14] and is a growing public health concern as it is often prepared in cafes and restaurants. 

The shelf life of a food product is often dependent on its pH, storage temperature, and water activity [15]. These factors are also important in controlling the growth and survival of pathogenic microorganisms in food products [16]. There are guidelines for food handlers and chefs to follow while preparing raw egg products like mayonnaise. According to the Australia New Zealand Food Standards Code, the pH of the raw egg products should be adjusted to 4.2 or below and should be immediately refrigerated to a temperature below 5 °C [17]. The document further states that the raw egg product should be made in small batches to minimize the time the raw egg product is at room temperature and all the processes in preparing the raw egg product such as “receipt, processing, storage, and display” should be performed at a temperature below 5 °C.

The internal and external pH both have the ability to control the biological and physiological processes of a bacterial cell [18], and unfavorable pH conditions can lead to loss of viability [19]. Interestingly, *Salmonella* utilizes mechanisms to survive unfavorable pH conditions and can achieve pH homeostasis [20]. This is facilitated by the cellular proton pumps, potassium/proton, and sodium/proton antiport systems [21], which enables the cell to be protected against acid stress [22]. Additionally, the acid tolerance response (ATR) of *Salmonella* also acts as an additional protective barrier against the antibacterial activity of the acids [18]. The ATR protects *Salmonella* spp. at low pH levels (pH 3 to 4), but is activated when the environmental pH values are between 6.0 and 5.5 and when pH homeostasis fails [18]. Due to these distinct characteristics, effectively controlling *Salmonella* has become a complex process. Moreover, pH also plays an important role in the structure and the stability of mayonnaise, which is an emulsion mainly consisting of eggs, oil, and vinegar. The isoelectric pH of the egg yolk proteins is an essential quality to consider because it affects the viscoelasticity and stability of the mayonnaise by facilitating the emulsification process [9]. 

The interaction between storage temperature and pH are likely the main factors that influence the survival of *Salmonella* in on-site prepared raw egg mayonnaise. Despite the availability of numerous guidelines for the control of *Salmonella* in raw egg products, the incidence of salmonellosis outbreaks associated with these products in Australia is still increasing. This is the first study to investigate the combined effect of pH and temperature on the survival of *S*. Typhimurium in on-site prepared raw egg mayonnaise with the aim of informing Australian food safety regulation.

## 2. Methods 

### 2.1. Culture

Two strains of *Salmonella enterica* serovar Typhimurium (ST) were used for the study, a standard *S*. Typhimurium strain from ATCC 53647 (STs) and a wild type *S*. Typhimurium (STi) strain which was isolated from a fecal sample of an infected individual. 

### 2.2. Survival of Salmonella in Acidic Peptone Water

For the experiment, the pH of peptone water (PW) (Oxoid Ltd., Basingstoke, Hampshire, England) was adjusted using acetic acid to attain a pH of 4.2, 4.4, or 4.6. The PW suspensions were incubated (Memmert incubator, Germany) at 37 °C, 23 °C, and 4 °C. PW at pH 7 incubated at 37 °C was used as the control. Each stage of the experiment was completed in triplicate, and the experiment was repeated three times.

The *Salmonella* strains were retrieved from the −80 °C freezer by inoculating on to Xylose Lysine Deoxycholate (XLD) agar (Oxoid Ltd., Basingstoke, Hampshire, England) plates using the streak plate method followed by incubation overnight at 37 °C. A typical *Salmonella* colony, with a black center, was introduced into 100 mL of PW pH 7 and was incubated at 37 °C overnight. The culture was centrifuged at 1500 g for 15 min, and the pellet was washed with PW and was resedimented by centrifugation at 1500 g for 15 min. 

The washed pellet was resuspended in PW, and the optical density at 600 nm (OD_600_) was adjusted to 1 which was approximately equivalent to 10^9^ CFU/mL [23] (UV-1800, Shimadzu UV-spectrophotometer, Japan) and was subsequently serially diluted to a suspension of 10^5^ CFU/mL which was used for the experiment. One (1) mL from this dilution was added to 100 mL of PW at pH 7, 4.6, 4.4, and 4.2. These cultures were each incubated at 37 °C, 23 °C, and 4 °C for 10 days. The drop plate method was used to plate 10 µL of each culture after every 2 h, 4 h, 6 h, 1 day, 2 days, 7 days, and 10 days to check the viability of the *Salmonella* on XLD agar. The number of representative colonies was recorded. 

### 2.3. Resuscitation of Salmonella Following Acid Treatment

The potential to resuscitate STi from a subset of incubations at different temperatures and pH conditions was determined. After 1, 2, 7, and 10 days of incubation at each pH and temperature, 1 mL from each of the samples was added to 9 mL of buffered peptone water (BPW) at pH 7 (Oxoid Ltd., Basingstoke, Hampshire, England) which was incubated at 37 °C overnight. Samples with increased turbidity were confirmed by spectrophotometric readings (UV-1800, Shimadzu UV-spectrophotometer, Japan) at 600 nm and confirmed to be *Salmonella* by plating on XLD agar. 

### 2.4. Survival of STi in Mayonnaise 

The recipe for the preparation of mayonnaise was adopted from the certificate III commercial cookery, student recipe learning guide from the Tertiary and Further Education training provider, South Australia (TAFE SA) [24]. All the steps were carried out aseptically under a biosafety hood. The eggshells were disinfected by wiping with 70% ethanol and then broken and yolks separated using an egg yolk separator. Five mL vinegar was added to six egg yolks and was mixed in a sterile laboratory blender (Waring commercial laboratory blender, USA) until a uniform suspension was obtained. Sunflower oil was added gradually while blending. 

The pH was tested (Hach Pocket Pro Multi 2, Colorado, USA) and was adjusted to pH 5.7 vinegar. To adjust the pH further to 4.2, 4.4, and 4.6, white vinegar was gradually added to the mayonnaise. The mayonnaise samples from each pH (5.7, 4.6, 4.4, and 4.2) were inoculated with STi to achieve a final concentration of 10^7^ CFU/g and were homogenized using a stomacher (Seward laboratory blender stomacher 400) for 2 min. Mayonnaise at pH 5.7 uninoculated with ST was prepared simultaneously and used as the negative control. 

Fifty grams of mayonnaise at each pH, inoculated with ST and the uninoculated mayonnaise were incubated at 37 °C, 23 °C, and 4 °C. Following each incubation time, the interval (0, 1, 2, 5, 7, and 10 days) mayonnaise was homogenized in the stomacher for 2 min, and 1 g of mayonnaise was mixed with 9 mL of 1% buffered peptone water (BPW). This step was performed in triplicate. From each of these triplicates, 10 µL was plated on to the XLD agar using the drop plate method, which was also performed in triplicate. The colonies with typical *Salmonella* colony morphology on XLD agar were counted and recorded. 

### 2.5. Resuscitation of Salmonella Following Incubation in Mayonnaise

At each sampling time point, BPW-mayonnaise mixtures were incubated at 37 °C overnight [25]. The incubated BPW-mayonnaise mixture was plated on XLD agar following an overnight incubation and was incubated at 37 °C overnight to check for resuscitation. 

### 2.6. Statistical Analysis

Repeated measures ANOVA was performed on SPSS software (IBM Corp. Released 2017. IBM SPSS Statistics for Windows, Version 25.0. Armonk, NY: IBM Corp.) to analyze the results and generate the graphs. 

## 3. Results

### 3.1. Acid Tolerance of STs and STi in Peptone Water (PW) under Different Temperature Conditions

**Figure 1:**
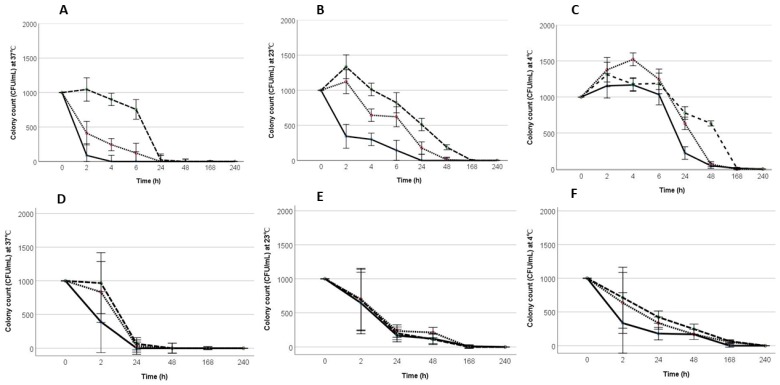

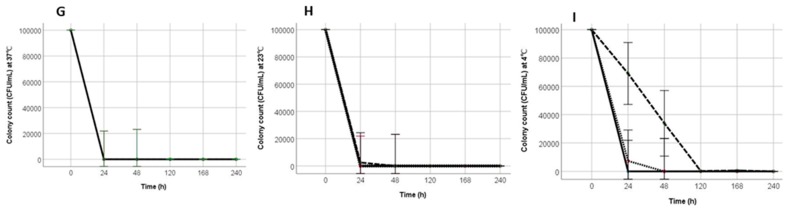
Survival of *S*. Typhimurium standard strain (STs) in peptone water (PW) at different pH and temperature conditions. (**A**–**C**) Survival of wild type *S*. Typhimurium (STi) in PW at 37 °C, 23 °C and 4 °C in different pH conditions, respectively. (**D**–**F**) Survival of STs in PW at 37 °C, 23 °C, and 4 °C, respectively. (**G**–**I**) Survival of STi in mayonnaise at 37 °C, 23 °C, and 4 °C. Error bars at a 95% confidence interval; n = 27; experiments were conducted in triplicate with the triplicate drop plate analysis for each). On the line graphs: “____ “indicates pH 4.2, “……” indicates pH 4.4, and “- - - - “indicates pH 4.6.

Acid tolerance of the STs and STi were determined at different incubation temperatures. Incubation at 4 °C significantly (*p* < 0.05) increased the survival of both ST strains under all acidic pH conditions (pH 4.2, 4.4, and 4.6), compared to incubation at 23 °C and 37 °C. The bactericidal effect of the acidic pH was most effective after 24 h of incubation at 37 °C. There were no viable STi at pH 4.2 at 37 °C (Figure 1A,D,G). Incubation at pH 4.6 was the least effective pH for inhibiting growth at all the temperatures tested. STs and STi, both showed exponential growth in the control PW cultures (pH 7, 37 °C or 23 °C). There was no growth observed when STs or STi were incubated at 4 °C at pH 7, but *Salmonella* survived at the low temperature for more than 10 days in both PW and mayonnaise.

### 3.2. Resuscitation of Salmonella Following Incubation at Acidic pH in PW

Although there were no observable colonies on the plates following incubation at 37 °C, at pH 4.4 and 4.6, resuscitation of STi was observed at pH 4.4 during the first 24 h in PW. However, resuscitation of STi was comparatively reduced at 37 °C (Table 1). Recovery of STi was observed following two days of incubation in pH 4.2 and pH 4.4 at 23 °C. At 4 °C, STi recovery was observed at all acidic pH conditions (pH 4.2, 4.4, 4.6) after 10 days of incubation, indicating the maximum survival at 4 °C. Complete and rapid inactivation of STi was observed at 37 °C at pH 4.2, and resuscitation was not possible. 

### 3.3. Survival and Resuscitation of STi in Mayonnaise

STi had a similar pattern of survival in mayonnaise (Figure 1G–I), as seen in PW. Maximum survival was observed at 4 °C in pH 4.6, and the viability was lost rapidly at 37 °C at pH 4.2 with no resuscitation (Table 2). STi survived for more than 10 days at pH 5.7, and there was no growth of STi in the mayonnaise, used as the negative control. At 23 °C, STi survived for more than 24 h at pH 4.6 with positive resuscitation after 48 h. No growth or resuscitation was observed at pH 4.2 and pH 4.4 following 24 h of incubation at 23 °C. Similarly, there was no growth or resuscitation observed after 24 h at 37 °C at pH 4.2, 4.4, or 4.6. In contrast, at 4 °C, resuscitation of STi were observed at a pH of 4.6, even after 10 days of incubation. Though the culturability of STi was lost following 24 h of incubation at pH 4.2 at 37 °C, when incubated at 4 °C, resuscitation was observed even after 10 days. 

## 4. Discussion

Ensuring that eggs and raw egg products are prepared, transported, and stored appropriately are crucial steps in reducing salmonellosis. For the preparation of raw egg mayonnaise, pH, and temperature are key factors for the control of *Salmonella*. Results of this study indicate that the combined effect of pH 4.2 and the incubation temperature, 37 °C was the most effective at reducing *S.* Typhimurium; however, incubating at 23 °C was still more effective than 4 °C and may present a more practical approach. The STi strain survived longer than the STs strain in the pH adjusted PW at 4 °C. The STi strain survived in pH 4.6 mayonnaise for more than 10 days at 4 °C, but viability was lost within 24 h at the same pH when stored at 37 °C. The long-term survival of *Salmonella* at 4 °C under acidified conditions also demonstrates the potential risk posed by cross-contamination in the refrigerator, cooking utensils, other ingredients, etc., and the importance of sanitation and cleaning. 

The lag phase is the earliest and the most poorly understood stage of the bacterial growth cycle [26]. It is presumed that during the lag phase, bacterial cells undergo an adaptation process required for survival in new environmental conditions [27]. The metabolic functions of a bacterial cell are also influenced by the growth environment [28]. These adaptive mechanisms, as well as the ability to achieve pH homeostasis, the ATR and the presence of a capsule makes *Salmonella* more resistant to environmental stresses [29]. Therefore, precisely engineered combinations of control mechanisms are needed to control the growth of *Salmonella*. 

These results differ from the Australian guidelines for the preparation of raw egg mayonnaise, which states that the pH should be adjusted to 4.2, and the raw egg products should be stored below 5 °C [17]. The guidelines further indicate that mayonnaise should be made in small batches every day, and the two-hour/four-hour rule should be maintained. This study showed that *S.* Typhimurium present in acidified mayonnaise (pH < 4.6) decreased over time, and that older batches of mayonnaise may present a lower risk for salmonellosis compared with a batch made that day. On the other hand, the advantage in making mayonnaise in small batches is that if a batch of mayonnaise is contaminated, then fewer people will be exposed to that particular batch. 

The pH of mayonnaise is reduced through the addition of vinegar and lemon juice [30]. The lipophilic nature of the undissociated acetic acid molecules penetrate the cell membrane and decrease the internal pH causing damage and death to the bacterial cells [31]. The antibacterial activity of the acid also depends on the storage temperature and the water activity (a_w_) of the egg-based sauce [32]. Higher temperatures rapidly reduced the viability of *Salmonella* when incubated at acidic pH. This is probably due to the increased cell membrane penetration of organic acids at higher temperatures [13]. Other variables that can affect *S.* Typhimurium survival in mayonnaise include the type of oil, garlic, spices, and plant essential oils (mint, cardamom, cinnamon, clove, orange, lemon, and grapefruit) [33]. Further research is needed to explore the combined effect of these different additions in recipes. 

The risk of storing mayonnaise at 37 °C is that if the pH is not correctly measured, the warmer temperatures will promote the growth of *S.* Typhimurium. The results of this study confirmed that at higher pH (i.e., pH 5.7 in mayonnaise and 7 in peptone water), there was rapid growth and increased cell numbers of *S.* Typhimurium at 37 °C and at room temperature; whereas, no growth was seen at 4 °C. This demonstrates the necessity for food handlers and chefs to confirm pH during the preparation of raw egg mayonnaise. 

## 5. Conclusions

This study showed that lower temperatures reduce the antibacterial activity of the organic acids, which allowed *Salmonella* to survive at low pH for longer. Preparing home-made mayonnaise at pH 4.2 and retaining at room temperature for at least 24 h effectively killed *S.* Typhimurium and could aid in reducing the prevalence of the salmonellosis outbreaks in Australia.

## 6. Further Research

Non-lethal but harsh environmental conditions could induce adaptive acid tolerance in *Salmonella*. Further studies into the development of acid tolerance of *Salmonella* in mayonnaise at different storage temperatures and pH conditions would be beneficial for further controlling salmonellosis outbreaks. As different species of *Salmonella* have different capacities to tolerate different environmental stressors, future research is needed to examine the survival of different sub-species of *Salmonella* in different pH and temperature conditions. The combined effect of garlic, salt, plant essential oils and spices in mayonnaise on the survival of *S.* Typhimurium should be assessed. There is also the need for further research to investigate the survival of other foodborne pathogens, such as *Campylobacter* spp., *Shigella* spp., and *E. coli*, in raw egg mayonnaise under these conditions prior to making a change in food safety regulations. 

## Figures and Tables

**Table 1:** Resuscitation (R) of wild type *S*. Typhimurium (STi) over time when incubated at different pH and temperatures in peptone water (PW).

Temperature (°C)	Day	pH 4.2	pH 4.2	pH 4.4	pH 4.4	pH 4.6	pH 4.6
		Average Colony Count on XLD Agar (CFU/mL)	Resuscitation/Growth	Average Colony Count on XLD Agar (CFU/mL)	Resuscitation/Growth	Average Colony Count on XLD Agar (CFU/mL)	Resuscitation/Growth
37	1	0	-	0	R	900	NA ^a^
37	2	0	-	0	-	600	NA ^a^
37	7	0	-	0	-	0	R
37	10	0	-	0	-	0	-
23	1	0	R	200	NA ^a^	400	NA ^a^
23	2	0	R	0	R	200	NA ^a^
23	7	0	-	0	-	0	R
23	10	0	-	0	-	0	-
4	1	200	NA ^a^	700	NA ^a^	0	R
4	2	100	NA ^a^	100	NA ^a^	0	R
4	7	0	R	0	R	0	R
4	10	0	R	0	R	0	R

^a^ If the Xylose Lysine Deoxycholate (XLD) agar plates had a positive growth for *Salmonella* the resuscitation/enrichment step was not conducted. 0 = No culturable cells above the limit of detection (10 CFU/mL). R = resuscitation of viable but nonculturable cells (VBNC) or enrichment to increase the concentration above the limit of detection.

**Table 2:** Resuscitation and survival of wild type *S*. Typhimurium (STi) over time when incubated at different pH and temperatures in mayonnaise.

Temperature (°C)	Day	pH 4.2	pH 4.2	pH 4.4	pH 4.4	pH 4.6	pH 4.6
		Number of Positives for Each of the Three Trials Conducted in Triplicate on XLD Agar ^a^	Number of Positives for Each of the Three Trials Conducted in Triplicate in Broth ^b^/R	Number of Positives for Each of the Three Trials Conducted in Triplicate on XLD Agar ^a^	Number of Positives for Each of the Three Trials Conducted in Triplicate in Broth ^b^/R	Number of Positives for Each of the Three Trials Conducted in Triplicate on XLD Agar ^a^	Number of Positives for Each of the Three Trials Conducted in Triplicate in Broth ^b^/R
37	0	27/27	NA	27/27	NA	27/27	NA
37	1	0/27	0/9	0/27	0/9	0/27	0/9
37	2	0/27	0/9	0/27	0/9	0/27	0/9
37	5	0/27	0/9	0/27	0/9	0/27	0/9
37	7	0/27	0/9	0/27	0/9	0/27	0/9
37	10	0/27	0/9	0/27	0/9	0/27	0/9
23	0	27/27	NA	27/27	NA	27/27	NA
23	1	0/27	0/9	0/27	0/9	9/27	8/9
23	2	0/27	0/9	0/27	0/9	0/27	3/9
23	5	0/27	0/9	0/27	0/9	0/27	0/9
23	7	0/27	0/9	0/27	0/9	0/27	0/9
23	10	0/27	0/9	0/27	0/9	0/27	0/9
4	0	27/27	NA	27/27	NA	27/27	NA
4	1	0/27	2/9	9/27	9/9	27/27	NA
4	2	0/27	6/9	0/27	5/9	19/27	9/9
4	5	0/27	5/9	2/27	6/9	14/27	7/9
4	7	0/27	0/9	0/27	3/9	9/27	4/9
4	10	0/27	1/9	0/27	0/9	0/27	4/9

^a^ Three trials were conducted; each trial was done in triplicate and with triplicate drop plate analysis for each NA = If there was growth observed on the drop plate then the resuscitation/enrichment step was not conducted/^b^ When there were no culturable cells above the limit of detection a resuscitation step was conducted. There were three trials conducted in triplicate with three broth resuscitation analyses conducted for each R—Resuscitation (which could also be increased in concentration to above the limit of detection.
